# Meningitis due to *Moraxella nonliquefaciens* in a paediatric patient: a case report and review of the literature

**DOI:** 10.1099/jmmcr.0.005086

**Published:** 2017-02-28

**Authors:** Carol Kao, Wendy Szymczak, Iona Munjal

**Affiliations:** ^1^​ Department of Pediatrics, Montefiore Medical Center, Albert Einstein College of Medicine, 111 E 210th St, Bronx, NY 10467, USA; ^2^​ Department of Pathology, Montefiore Medical Center, Albert Einstein College of Medicine, 111 E 210th St, Bronx, NY 10467, USA

**Keywords:** *Moraxella nonliquefaciens*, meningitis, Crouzon syndrome, recurrent meningitis

## Abstract

**Introduction.** *Moraxella nonliquefaciens* is an unusual organism to be isolated from cerebral spinal fluid (CSF) and there exists only one case report of *M. nonliquefaciens* meningitis from a neonate. *Moraxella* species normally exist as part of the human upper respiratory tract flora and rarely cause invasive human disease. There are only a handful of case reports implicating the organism as a cause of endocarditis, bacteraemia, septic arthritis and endophthalmitis. Identification to the species level based on routine laboratory techniques has been challenging, with final identification often made through 16S rRNA sequencing. With the use of a newer diagnostic tool, matrix-assisted laser desorption ionization-time of flight (MALDI-TOF) MS, we were able to rapidly identify the organism and initiate appropriate treatment.

**Case presentation.** We present a rare care of *M. nonliquefaciens* meningitis in a paediatric patient with an underlying cranial anatomical defect due to Crouzon syndrome. She had been admitted to hospital 3 months previously with *Streptococcus pneumoniae* meningitis and mastoiditis, and returned to the emergency department with meningismus. CSF culture grew *M. nonliquefaciens*. She was treated with ceftriaxone with rapid improvement and eventually was taken for endoscopic surgical repair of a right encephalocele defect.

**Conclusion.** The use of MALDI-TOF MS allowed for the rapid identification of the organism. The patient recovered with appropriate antimicrobial therapy and eventual surgical correction. An underlying anatomical defect should be considered in all patients who present with meningitis due to this unusual organism.

## Abbreviations

CSF, cerebral spinal fluid; MALDI-TOF, matrix-assisted laserdesorption ionization-time of flight.

## Introduction

*Moraxella nonliquefaciens* is an aerobic, Gram-negative coccobacillus that is part of the normal flora of the human upper respiratory tract. The organism rarely causes invasive infections. We report a case of meningitis due to *M. nonliquefaciens* in a paediatric patient with an underlying cranial anatomical defect, which predisposed her to recurrent episodes of bacterial meningitis from organisms known to colonize the nasopharyngeal tract.

## Case report

We present the case of a 16-year-old female with Crouzon syndrome, who had undergone a Lefort III cranial reconstruction 5 years prior without any complications. She presented to the hospital emergency room with a 1 day history of fever, headache and neck stiffness. Symptoms had started the day prior at school and had worsened. The patient had travelled to Upstate New York (USA) the weekend prior and had stayed with other individuals in a wooden cabin, but denied swimming in freshwater lakes, denied wildlife contact and no other attendees had similar symptoms to her knowledge.

It is important to note that she had been admitted 3 months prior for similar symptoms and was diagnosed with acute bacterial meningitis, mastoiditis and bacteraemia due to *Streptococcus pneumoniae*. At that time, she was treated with ceftriaxone, followed by an outpatient course of levofloxacin for a total of 6 weeks. An magnetic resonance imaging (MRI) ofher head was obtained during that hospitalization and was significant for a right ethmoid meningocele, dehiscence of the right cribriform plate and fovea ethmoidalis. The Neurosurgery team was consulted and elected to defer surgical correction until acute infection had resolved. Prior to discharge, the patient received a MenACWY-D booster, a Tdap booster and annual inactivated flu vaccine, and her primary dose of PCV-13 vaccine with PPS-23 was given 2 months later in the hospital outpatient clinic due to the presence of an uncorrected central nervous system (CNS) defect.

## Investigations

The paediatric infectious disease team was called to the emergency room to evaluate a 16-year-old patient known to our service with Crouzon syndrome and a recent hospitalization. The patient was noted to be febrile, with a heart rate of 100 beats min^−1^, a respiratory rate of 24 breaths min^−1^,oxygen saturation of 99 % on room air and blood pressure 105/48 mmHg (14/6.4 kPa). Physical examination was significant for nuchal rigidity and photophobia. Laboratory tests revealed a peripheral white blood cell count of 13 500 cells µl^−1^ (86 % neutrophils), a haemoglobin level of 11.2 g dl^−1^ and a platelet count of 223 000 platelets µl^−1^. Lumbar puncture was performed and cerebral spinal fluid (CSF) studies showed an increased white blood cell count of 324 cells µl^−1^ (87 % neutrophils), with a glucose concentration of 46 mg dl^−1^ and a protein level of 102 mg dl^−1^. Routine blood chemistry results were within the reference range, including a blood glucose level of 93 mg dl^−1^. Blood culture, urine culture and a CSF culture were instigated, and the patient was started on cefepime 2 g IV Q8 and vancomycin 1 g IV Q8.

## Diagnosis

**Fig. 1.**
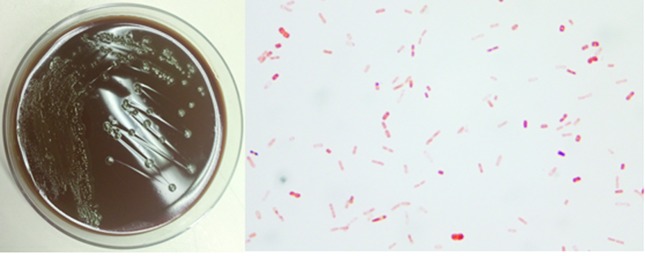
Left: growth of *M. nonliquefaciens* on a chocolate agar plate. Right: Gram staining of the organism.

After approximately 72 h incubation, flat, grey, 0.5 to 1 mm colonies with slightly spreading edges were observed on culture plates. Gram staining of the isolate revealed short, plump Gram-negative rods and diplococcus-like forms [Fig. 1]. The isolate was identified as *M nonliquefaciens* by matrix-assisted laser desorption ionization-time of flight (MALDI-TOF) MS and confirmed by DNA sequencing.

## Treatment

The isolate was found to be a β-lactamase producer. The patient's treatment was changed from cefepime and vancomycin to ceftriaxone 2g IV Q12 based on susceptibility testing in comparison to reported MICs in the literature, as there are no Clinical and Laboratory Standards Institute standard MIC values for *M. nonliquefaciens*. The patient improved rapidly and was taken for endoscopic surgical repair of the right encephalocele defect after completing a 2 week course of antibiotics.

## Discussion

*The* genus *Moraxella* is composed of oxidase-positive, non-motile, asaccharolytic, aerobic Gram-negative bacteria. Most species of *Moraxella* are fastidious, although growth can be enhanced by incubation in capnophilic conditions. Gram-stain morphology varies within the genus, with bacteria appearing as diplococci, coccobacilli or short rods. *Moraxella* spp. are part of the normal flora of the human respiratory and genital tracts, and are considered to have low pathogenic potential. The most common species causing infection in humans is *Moraxella catarrhalis*, which can cause otitis media in children and exacerbations of chronic obstructive pulmonary disease [1, 2]. Other *Moraxella* species capable of causing rare infections include *Moraxella atlantae*, *M. canis*, *M. catarrhalis*, *M. lacunata*, *M. lincolnii*, *M. nonliquefaciens* and *M. osloensis*, which was separated from *M. nonliquefaciens* in 1967*. Moraxella* sp. rarely cause invasive disease and only a handful of case reports exist implicating the organism as a cause of endocarditis, bacteraemia, septic arthritis, endophthalmitis and meningitis [3–8].

We reviewed the literature on *Moraxella* sp. as a cause of meningitis. One case report described *M. osloensis* meningitis secondary to extensive sinusitis leading to erosion through the cribriform plate, and another case series of three patients with *M. osloensis* meningitis included two paediatric patients where a predisposing factor could not be identified [3, 4]. Only one case of *M. nonliquefaciens* meningitis has been reported in a neonate from 1982 [9]. In our particular patient, her cranial defect most likely predisposed her to recurrent meningitis with nasopharyngeal organisms, and the presence of an underlying anatomical defect should be considered in patients who present with meningitis due to *Moraxella* sp.

In the previously reported cases of *M. osloensis* meningitis, the isolates could not be identified to the species level based on routine laboratory techniques such as the API ID 32 GN system and the Vitek2 GN system (bioMérieux), and the final identification was made through 16S rRNA gene sequencing. With the introduction of MALDI-TOF, we were able to quickly identify *M. nonliquefaciens* as the causative agent. The ability of MALDI-TOF to identify *Moraxella* sp. and other fastidious organisms has been evaluated elsewhere [10]. MALDI-TOF was able to accurately identify isolates of *M. nonliquefaciens* and other species of *Moraxella* [10]. The rapid identification of the bacteria in our case ensured that our patient was receiving appropriate therapy based on published antibiotic susceptibility data.

*Moraxella* species are typically susceptible to penicillin, cephalosporins, quinolones, aminoglycosides and macrolides. *M. nonliquefaciens* is not commonly implicated in human disease so detailed susceptibility patterns are not fully known, although it is reportedly susceptible to penicillin, ampicillin, ceftazidime, tetracycline, ciprofloxacin, gentamicin, erythromycin and imipenem [11, 12]. A recent study reported increased resistance in clinical strains to macrolides [13]. Our isolate was β-lactamase positive with a low MIC value for ceftriaxone, generally considered susceptible.

In conclusion, meningitis due to *M. nonliquefaciens* is exceedingly rare. We report a unique paediatric case where an underlying CNS defect predisposed to recurrent meningitis with organisms that routinely inhabit the nasopharyngeal flora and are not typically implicated in invasive disease.
